# Knowledge and Attitudes about Transcranial Magnetic Stimulation among Psychiatrists in Oman: A cross sectional study

**DOI:** 10.1192/j.eurpsy.2023.575

**Published:** 2023-07-19

**Authors:** M. K. Albalushi

**Affiliations:** psychiatry, Oman medical specialty board, Muscat, Oman

## Abstract

**Introduction: Background and Objective:**

Repetitive transcranial magnetic stimulation (rTMS) is a noninvasive treatment method and the only been approved for medication refractory depression by US Food and Drug Administration (FDA). However, comprehensive knowledge about rTMS is not yet widespread among psychiatrists. The aims of this study were to assess psychiatrists’ knowledge of and attitudes toward rTMS and to determine the contributing factors on knowledge of rTMS in Oman.

**Objectives:**

A quantitative observational cross-sectional study will be conducted using an online survey. Demographic information, knowledge of and attitudes towards rTMS measures were collected. Both univariate analysis, multiple linear regression was performed to identify the risk factors associated with knowledge levels.

**Methods:**

A quantitative observational cross-sectional study will be conducted using an online survey. Demographic information, knowledge of and attitudes towards rTMS measures were collected. Both univariate analysis, multiple linear regression was performed to identify the risk factors associated with knowledge levels.

**Results:**

A total of 50 psychiatrists participated in this study (response rate = 83%). The average age of the participants is 32.7±4.3 years [26.0-41.0], more than half of them were females (n=28, 56.0%), and resident (Junior/Senior) (n=25, 50.0%). The majority of the samples are Omani (n=45, 90.0%), working in the tertiary hospital (n=38, 76.0%). The average scores on knowledge of and attitudes towards rTMS in this sample were 14.5±3.8 and 22.5±6.3, respectively. Linear model showed that senior residents and above had a higher knowledge level than junior residents (ß=4.65, p<.001). Those samples with the rTMS device in their work-place had a higher knowledge level than don’t have (ß=1.88, p=0.027).

**Conclusions:**

Three factors have a directional effect on the level of knowledge among psychiatrists toward rTMS, namely, higher educational level, presence of rTMS device at the workplace and availability of standardized training in Rtms.

**Disclosure of Interest:**

None Declared

